# An assessment of Ghana’s pilot of the RTS,S malaria vaccine implementation programme; 2019–2021: a retrospective study

**DOI:** 10.1186/s12936-024-05113-8

**Published:** 2024-09-27

**Authors:** Michael Rockson Adjei, Peter Ofori Tweneboah, George Bonsu, Janet Vanessa Baafi, Kwame Amponsa-Achiano, Franklin Asiedu-Bekoe, Sally-Ann Ohene, Patrick Kuma-Aboagye, Martin Peter Grobusch

**Affiliations:** 1 Center of Tropical Medicine and Travel Medicine, Department of Infectious Diseases, Amsterdam University Medical Centers, Location AMC, University of Amsterdam, Amsterdam, The Netherlands; 2 World Health Organization, Country Office, Accra, Ghana; 3 PATH, Accra, Ghana; 4 Ghana Health Service, District Health Directorate, Sunyani West, Odumase, Ghana; 5 Ghana Health Service, Headquarters, Accra, Ghana; 6 Institute of Tropical Medicine, and German Center of Infectious Diseases (DZIF), University of Tuebingen, Tuebingen, Germany; 7 Institute of Infectious Diseases and Molecular Mediicne, University of Cape Town, Cape Town, South Africa; 8 Centre de Recherches Médicales en Lambaréné (CERMEL), Lambaréné, Gabon; 9 Masanga Medical Research Unit, Masanga, Sierra Leone

**Keywords:** RTS,S, R21, Malaria vaccine implementation programme, Post-introduction evaluation, New vaccine introduction checklist, Ghana

## Abstract

**Background:**

In May 2019, Ghana piloted the introduction of RTS,S malaria vaccine into routine immunization in 42 districts of seven of the 16 regions. The RTS,S malaria vaccine implementation programme (MVIP) post-introduction evaluation (PIE) conducted in Ghana, assessed the immunization system as well as healthcare worker and caregiver experiences during the phase-one rollout but was less expressive on quantitative grading of the respective thematic areas of the vaccine introduction plan. Given the utility of summary statistics in programme evaluation and communication, this follow-up study aimed to provide an overall rating of the country's performance regarding the MVIP .

**Methods:**

A retrospective study was conducted from 10th January to 5th February 2024. It involved review of records to assess key thematic areas of the national MVIP plan, using a study tool adapted from the WHO New Vaccine Introduction (NVI) checklist. A composite score ranging from zero to 100 per cent was generated to assess the country's overall performance regarding introduction of the malaria vaccine, rated on a Likert scale as comprehensive, good, fair, and poor.

**Results:**

The overall performance in the MVIP was rated 78.9% (30/38) corresponding to a grading of “good” on the Likert scale. Performance indicators under thematic areas including policy, national coordination mechanisms, waste management, health worker training, and pharmacovigilance were completely achieved. However,  some weaknesses were exhibited in areas such as financial consideration, cold chain, logistics, and vaccine management, and monitoring and evaluation.

**Conclusion:**

Ghana’s MVIP demonstrated remarkable strengths worth leveraging  to improve the national immunization programme. The weaknesses observed in some of the thematic areas present opportunities to engage key immunization partners and stakeholders towards aligning efforts to ensure a more robust expansion phase. The lessons from the MVIP may be relevant to areas introducing malaria vaccine irrespective of the product type—RTS,S or R21.

**Supplementary Information:**

The online version contains supplementary material available at 10.1186/s12936-024-05113-8.

## Background

Malaria, a life-threatening but preventable and curable disease is caused by protozoan *Plasmodium falciparum* transmitted through the bite of female *Anopheles* mosquito. The disease can be mild, but life-threatening manifestations including severe anaemia, acute renal failure, and cerebral malaria may occur among susceptible individuals [1].

Globally, there were an estimated 247 million cases in 2021 compared with 245 million in 2022 [2]. The majority (95%) of the cases were recorded in the World Health Organization (WHO) African Region with four countries (Nigeria, Democratic Republic of Congo, Uganda, and Mozambique) accounting for almost half of the global cases. Despite the increase in cases, global malaria deaths have declined steadily from 30.1 per 100,000 in 2000 to 14.8 per 100,000 in 2021. In the WHO African Region, malaria deaths decreased from 148 per 100,000 to 58 per 100,000 within the same period [2] Children under five years are among the most-vulnerable and account for about 80% of malaria related mortalities globally [3].

Malaria is both endemic and perennial throughout Ghana, putting the entire population at risk. In 2021, the WHO projected that there were an estimated 5.3 million malaria cases with 12,500 estimated deaths recorded [4]. About 30% and 23% of outpatient and inpatient attendances, respectively, are due to malaria [4]. The country has made significant advancement in the control of the disease through deployment of multiple interventions including, among others, seasonal malaria chemoprevention (SMC), indoor residual spraying (IRS), larval source management (LSM), intermittent preventive treatment in pregnancy (IPTp), distribution of long-lasting insecticidal nets (LLIN), and optimized case management (RDT/ACT) [5, 6]. These efforts are aligned with the Sustainable Development Goals targets of reducing malaria incidence and mortality by at least 90% respectively, by 2030 [7].

Significant investment has been made into  global malaria prevention, and research to innovate new tools and scale up interventions to facilitate disease elimination are ongoing. In 2015, the WHO issued a position paper calling for large-scale pilot implementation of RTS,S malaria vaccine to be delivered alongside other malaria interventions in settings of moderate to high parasite transmission in sub-Saharan Africa [8]. With malaria being a significant cause of morbidity especially among children in Ghana, the country’s expression of interest to pilot the vaccine introduction was approved by the WHO in 2017, alongside Malawi and Kenya. The addition of a vaccine to the malaria control interventions affirms the global commitment towards eliminating the disease. In May 2019, Ghana piloted the introduction of RTS,S malaria vaccine into routine immunization in 42 districts in seven regions [9]. Following the successful outcome of the pilot, the WHO recommended RTS,S malaria vaccine for wider use in endemic settings prioritizing areas of moderate to high transmission [10].

Funding and vaccine availability are key determinants of the pace of rollout of malaria vaccine, even in the pilot countries. Recent developments at the global level, notably GAVI commitment of additional resources towards malaria vaccine introduction in 2023–2025 [11]; and pre-qualification of R21 malaria vaccine by WHO give leverage to the global malaria elimination drive [12]. These events are expected to improve access to financial and technical support for high-burdened countries and boost global vaccine supply to facilitate introduction into national immunization programmes.

Post introduction evaluation (PIE) of new vaccine provides vital lessons to improve existing programmes and strengthen new introductions. Given the growing interest in the malaria vaccine globally, a comprehensive documentation of the RTS,S malaria vaccine implementation programme (MVIP) pilot experiences could serve a valuable resource to new areas introducing the vaccine. The RTS,S MVIP PIE conducted in Ghana, assessed the immunization system as well as healthcare worker and caregiver experiences during the phase-one plan, but was less expressive on quantitative grading of the respective thematic areas of the national vaccine introduction plan [9]. Given the utility of summary statistics in programme evaluation and communication, this follow-up study aimed to provide an overall rating of the country's performance regarding the MVIP.

## Methods

### Study design

A retrospective study was conducted from 10th January to 5th February 2024. It involved review of records to assess key thematic areas of national malaria vaccine introduction plan, using a tool adapted from the WHO New Vaccine Introduction (NVI) checklist Vaccine [13].

### Selection of MVIP pilot areas

**Fig. 1:**
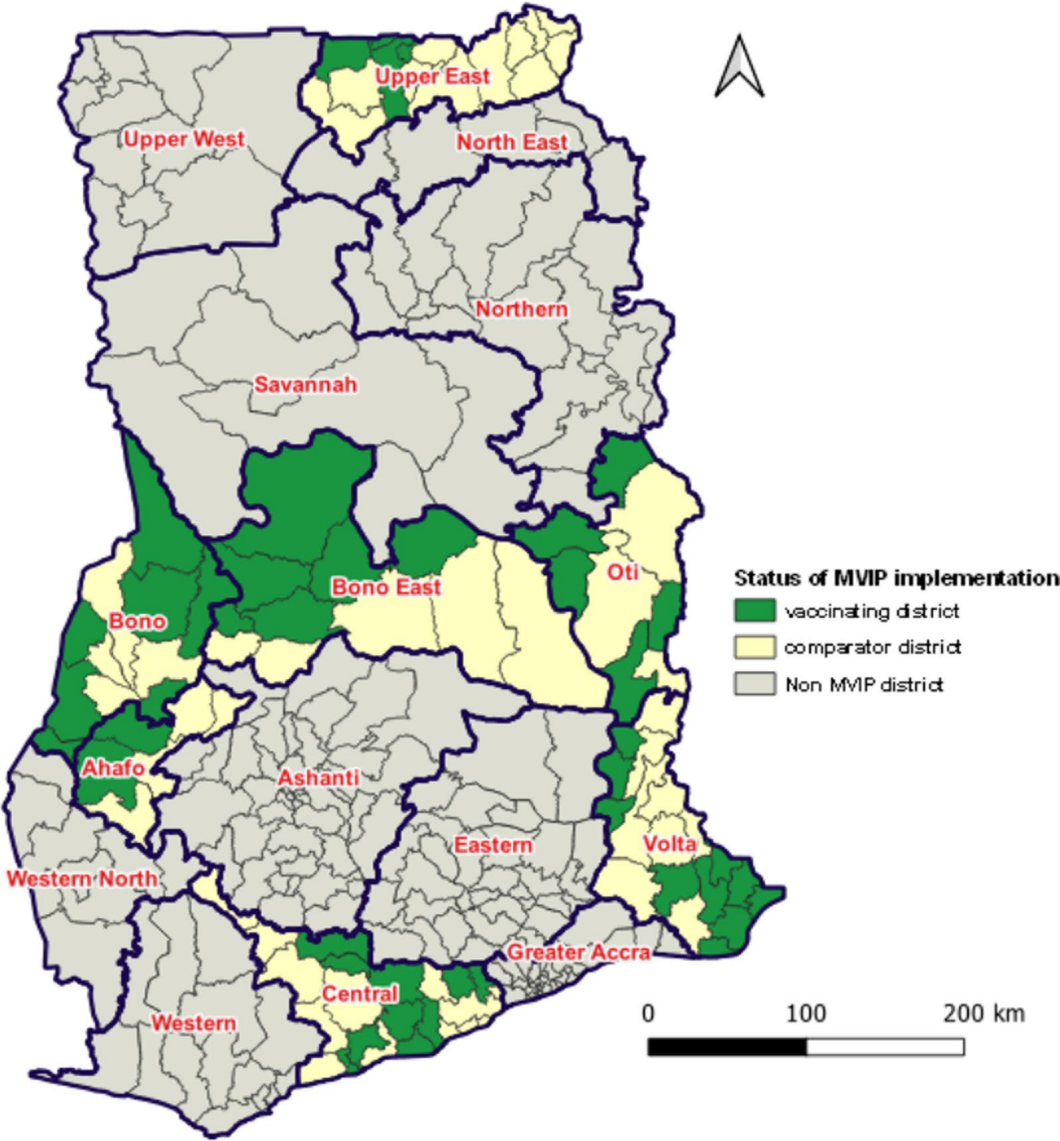
Malaria vaccine implementing regions, Ghana. Source: Adjei et al*.* [9]

Ghana has sixteen regions (Fig. 1) and three climate zones namely Savannah, Forest, and Coastal zones classified according to rainfall; relative humidity; and maximum and minimum temperature records from 1976 to 2018 [14]. Malaria transmission varies from intense seasonal transmission in the Savannah zone to perennial in the other zones [15].

The malaria vaccine was deployed in 42 districts of seven regions in phase-one of the rollout plan (Fig. 1). The selection of the districts was based on high malaria burden; high immunization coverage; and high number of age-eligible children to receive the vaccine. The remaining districts in the implementing regions served as comparator areas to facilitate impact assessment.

### Data collection and analysis

#### Data source

Data was collected from two sources: the national MVIP plan, and the MVIP PIE  report. Review of the documents (data collection) and rating  of the thematic areas were conducted by an independent assessor from a public university in Ghana.

The RTS,S malaria vaccine introduction plan outlined how the country intended to introduce the malaria vaccine. The document was developed by a multi-disciplinary technical working group drawn from the Ministry of Health, Ghana Health Service, and health partners including WHO, PATH, and UNICEF. It elaborated the background and context of vaccine introduction; goals, objectives, expected impact and challenges; strategies and activities; and budget and financing [Ministry of Health/Ghana Health Service, RTS,S malaria vaccine introduction plan for Ghana, April 2018, unpublished].

The RTS,S malaria vaccine post introduction evaluation was conducted in 18 districts selected from the 42 vaccinating districts in the seven pilot regions, to assess implementation of the MVIP, and its impact on the immunization system. It involved interviews of key health staff and caregivers, review of documents, and observation of immunization service delivery and storage areas [9]. Survey districts were selected purposively, ensuring representation of every pilot region with consideration given to immunization coverage, residential settings, and geographical spread.

### Assessment and rating of thematic areas

The assessment tool was adapted from the WHO NVI checklist [13]. The NVI checklist was modified to include scores for the respective thematic areas of new vaccine introduction. The MVIP  was assessed by a composite rating system. The key activities or milestones regarding  the  RTS,S malaria vaccine introduction were to be captured in MVIP plan and implemented within 6–12 months of the pilot. An ‘all or none’ grading system was applied: planning or execution of a key activity attracted a score of one, and zero for the contrary. An activity that was captured in the MVIP plan and implemented accordingly attracted a composite score of two—one each for planning and implementation.

The total score in a thematic area depended on the number of key activities or milestones. This ranged from minimum of one (for programme objectives; policy; financial consideration; national coordination; and waste management) to a maximum of five (for Advocacy, Communication, and Social Mobilization). The maximum score for each arm (planning and implementation) was 19, giving a composite score of 38 for the expected overall rating. The score for the overall MVIP performance was expressed as a percentage and interpreted on the Likert scale as: (1) ≥ 90%: comprehensive; (2) 70–89%: good; (3) 50–69%: fair; and (4) < 50%: Poor.

The country’s score sheet was shared with selected key stakeholders involved in the MVIP for validation. Verbal attestations were not accommodated, and disagreements in scoring were resolved by referring to the source documents.

## Results

The composite scores were 15 each for planning and implementation, respectively (Table 1), with an overall rating of 78.9% (30/38) corresponding to “good” on the Likert scale.

**Table 1:** Thematic area ratings, MVIP Pilot, Ghana; 2019–2021

SNo	Thematic area	Rating		
		Planning	Implementation	Composite
1	Programme objective	1/1	1/1	2/2
2	Target population and delivery strategy	3/3	2/3	5/6
3	Policy	1/1	1/1	2/2
4	Financial consideration	0/1	0/1	0/2
5	National coordination mechanism	1/1	1/1	2/2
6	Cold chain, logistics, and vaccine management	0/1	1/1	1/2
7	Waste management	1/1	1/1	2/2
8	Monitoring and evaluation	2/2	1/2	3/4
9	Health worker training	2/2	2/2	4/4
10	Pharmacovigilance	1/1	1/1	2/2
11	Advocacy, communication, and social mobilization	3/5	4/5	7/10
	Overall	15/19	15/19	30/38

Below were the ratings in the respective thematic areas:

### Programme objective (Composite score: 2/2; 100%)

The MVIP sought to assess operational feasibility, safety, and impact of the RTS,S malaria vaccine in the context of routine use among children. These objectives were clearly documented in the vaccine introduction plan and remained in focus during the pilot. Primary and secondary data were collected as part of the implementation process to assess the programme objectives.

### Target population and delivery strategies (Composite score: 5/6; 83.3%)

The MVIP activities originally targeted up to 78 districts: 38–40 vaccinating, and 36–38 non-vaccinating. Although the total number of implementing areas remained within target, the allocation per category fell out of plan. The vaccinating districts were increased from 40 to 42 due to addition of two more districts from the Volta Region, while the non-vaccinating districts decreased to 34.

As was indicated in the rollout plan, the vaccine was introduced through a phased approach (priority given to areas with high malaria burden) targeting children 6–24 months. To reach all eligible children including those living in underserved communities, all health facilities in the vaccinating districts were to administer the vaccine as part of routine immunization package. However, the PIE indicated that, not all health facilities provided malaria vaccination.

### Policy (Composite score: 2/2; 100%)

With funding from partners, the immunization data collection tools comprising child health record books, Expanded Programme on Immunization (EPI) tally books, monthly reporting forms, and other reporting templates were revised to include sections for RTS,S malaria vaccine. Datasets in the District Health Information Management Systems (DHIMS) were also updated. Job aids were printed to facilitate decision making at service delivery points. These changes ensured integration of the vaccine into the childhood immunisation schedule.

### Financial considerations (Composite score: 0/2; 0)

There was no plan to mobilize local resources to support the MVIP. The direct cost of the vaccine rollout  was borne by partners, although government’s routine expenditure on health, such as payment of staff salary indirectly supported the vaccine deployment.

### National coordination mechanism (Composite score: 2/2; 100%)

The MVIP coordination mechanism was integrated into the existing governance structure of the health sector. The Interagency Coordinating Committee (ICC) under the co-chairmanship of the Chief Director of the Ministry of Health and the Director General of the Ghana Health Service (GHS) was responsible for the overall coordination of the pilot. MVIP Technical Working Group was formed with subcommittees responsible for resource mobilization; advocacy, communication, and social mobilization; training and service delivery; data management, monitoring and evaluation; logistics and waste management; and surveillance and safety monitoring.

### Cold chain, logistics, and vaccine management (Composite score: 1/2; 50%)

The existing cold chain capacity was deemed adequate and did not require expansion, except replacement of obsolete cold chain monitoring equipment. However, during the implementation, additional vaccine fridges were procured through the Cold Chain Equipment Optimization Platform (CCEOP) to improve availability at health facilities.

### Waste management strategy (Composite score: 2/2; 100%)

In anticipation of increased immunisation waste generation, additional incinerators were constructed in some regions and districts, and this was captured in the rollout plan.

### Monitoring and evaluation (Composite score: 3/4; 75%)

Monitoring and supervision of service delivery, as contained in the introduction plan was implemented at all levels of the health system to strengthen service delivery. Joint monitoring teams comprising of the GHS and partners provided periodic technical support to the subnational levels. The post-introduction evaluation expected to be conducted 6–12 months after pilot was carried out after 24 months (August-November 2021).

### Health worker training (Composite score: 4/4; 100%)

Training was planned and implemented using a downstream approach: national level training of trainers was conducted for national and regional level resource persons. Following this, cascade trainings were conducted at the district and subdistrict levels to build capacity of frontline health staff. Focal points were designated at the district and subdistrict levels with functions including orientation of newly posted staff on the malaria vaccine and monitoring of service delivery.

### Pharmacovigilance (Composite score: 2/2; 100%)

The safety sub-committee developed tools and guidelines to facilitate reporting of adverse events following immunization (AEFI) and adverse events of special interest (AESI). Capacity of health staff was built to strengthen reporting from service delivery points to the National Regulatory Authority, Food and Drug Authority (FDA).

### Advocacy, communication, and social mobilization (Composite score: 7/10; 70%)

National and sub-national launches of the RTS,S malaria vaccine were held prior to the rollout. Education materials were developed to facilitate delivery of key messages to the target population, including caregivers and health workers. Health workers led communication and demand generation activities, and community members (including opinion leaders, community-based volunteers) were brought on board after implementation had travelled a while.

Information was delivered in local languages via  radio, television, service delivery point, and information vans. The use of social media for information sharing was not considered in the MVIP plan, but became instrumental  in addressing rumours, misinformation, disinformation, and other public concerns during the rollout. Spokespersons were trained and designated at national and sub-national levels to address caregiver and community concerns.

## Discussion

The study summarizes Ghana’s performance in the RTS,S MVIP pilot, and brings to fore strengths and opportunities to improve future vaccine introductions. The country has a rich experience in new vaccine introductions dating back from inception of the immunization programme in 1978 [16]. Having started with vaccines against the so-called ‘six childhood killer diseases’ [17], the immunization portfolio has expanded progressively and as of December 2023, 11 vaccines against 14 vaccine preventable diseases (VPDs) including COVID-19 were administered in the national immunization programme [16]. It is instructive to note that the national immunisation programme primarily targets children under five years and women [16]. However, the advent of COVID-19 pandemic necessitated the adoption of life-course approach, making vaccines accessible to all persons across the various age groups [18].

Although the overall rating was ‘good’, a near perfect vaccine pilot programme with grading on the scale of ‘comprehensive’ was anticipated, given the country’s experience in new vaccine introductions [19, 20]. However, it is imperative to interpret the country’s performance in the context of other events or factors. Firstly, the botched Ebola vaccine trial in 2015 heightened public skepticism about new vaccines and immunisation [21] and might have cast a shadow over the malaria vaccine introduction. The proposed studies were suspended by the Ministry of Health of Ghana amid protest from members of parliament and the public, due to communication gaps on the rationale of the trial and safety of the Ebola vaccine [21]. Secondly, every vaccine introduction could present challenges that may not yield to innovations and experiences from past exercises, despite working well in previous vaccine introductions [19, 20, 22]. Thirdly, the assessment tool is being used first-time, hence relating the country’s MVIP performance to past vaccine introductions might be challenging due to contextual differences.

Despite the foregoing, the study observed strengths in key thematic areas including programme objective, policy, national coordination mechanisms, waste management, health worker training, and pharmacovigilance, that attest to the robustness of the national  immunisation programme [23]. Preparations towards the malaria vaccine introduction began ten years earlier (in 2009) with the formation of a Malaria Vaccine Technical Working Group as a subcommittee to the then National Malaria Control Programme (rechristened National Malaria Elimination Programme to reflect programme goal). The key function of the subcommittee was to compile and evaluate evidence on the use of the RTS,S malaria vaccine in Ghana [24]. This forethoughtfulness might have facilitated thorough planning and bolstered successes achieved in the RTS,S malaria vaccine pilot [25].

Strong stakeholder collaboration is critical in new vaccine introduction. A study conducted in Ghana by Omolola et al. (2023) on barriers and facilitators to nationwide implementation of the malaria vaccine, cited stakeholder involvement as a key driver for successful introduction and scale-up [24]. Recognition of roles of the multiple stakeholders might have facilitated early onboarding of key actors including the National Regulatory Authority–FDA, research institutions, and health partners to plan and implement the MVIP. Given that, the vaccine introduction was largely donor-funded, such collaboration was necessary to ensure mobilization of resources from key partners including GAVI, WHO, and PATH to facilitate implementation [26]. The stakeholder collaboration mechanism was fostered by the existence of an ICC at the Ministry of Health to coordinate health partner inputs [27]. The ICC provided the platform to bring together the key stakeholders to kick start preparatory works on the vaccine introduction.

The structure of Ghana’s health system partly accounted for the strengths observed in the RTS,S malaria vaccine introduction. Ghana runs a three-tier health system that is decentralized to the community level but operates seamlessly. Each tier has some level of autonomy to implement contextual health strategies with the aim of achieving the broader national objective [28]. This arrangement ensured that, implementation challenges encountered at the various levels were largely confined and quickly addressed to mitigate escalation. For instance, so pervasive was social media-aided spread of misinformation that, the national immunisation programme developed communication guidelines for adoption by the implementing regions and districts. This strengthened community engagement  in  addressing key  concerns [9].

The assessment also highlighted gaps that could be bridged to improve future vaccine introductions. Target population and delivery strategy; financial considerations; cold chain, logistics, and vaccine management; monitoring and evaluation; and advocacy, communication, and social mobilization were among the weak spots. Local funding was not mobilized to support the MVIP,  probably because of  assurance of donor resources [26]. On the other hand, attempts might have been made but this could not be proven, given the absence of documentary evidence. The non-implementation of planned activities suggests that, resources allocated for the MVIP  were inadequate and might have resulted in deprioritization of key activities. On the contrary, implementation of ad hoc activities might have led to repurposing of  resources and contributed to nonexecution of planned activities [29].

Involvement of local leaders in advocacy and the use of social media are essential in improving vaccine acceptance [30], although not prioritized in the MVIP  plan. However, during implementation, and in response to vaccine hesitancy community health volunteers led communication to assure vaccine safety, and social (new) media was leveraged to combat misinformation [9].

A limitation of the study was that, the assessment tool  was applied first-time to evaluate vaccine introduction in Ghana, and it is possible the ratings  were biased. To mitigate this, the tool was validated by local experts, and piloted in one of the MVIP districts to affirm reliability. Again, the use of only documentary evidence as proof of implementation might have led to underestimation of the country’s performance, given that activities implemented ad hoc were corroborated by some of the key informants, but were not taken into account  due to rigidity of the assessment tool. 

## Conclusion

This study assessed Ghana’s performance in the RTS,S malaria vaccine pilot and rated it as ‘good’. Programme objectives; policy; national coordination mechanisms; waste management strategy; health worker training; and pharmacovigilance were among strengths, while target population and delivery strategies; financial consideration; cold chain, logistics, and vaccine management; monitoring and evaluation; and advocacy, communication, and social mobilization present opportunities to improve future vaccine introductions. The lessons from the MVIP may be relevant to new areas introducing the malaria vaccine irrespective of the product type – RTS,S or R21.

Given that Ghana is expected to fully fund its immunization programme from 2030 [31], it is imperative to leverage local resources to foster ownership and sustain introduction of the malaria vaccine beyond the pilot areas. Non-Governmental Organizations and local partners may consider leading mobilization of resources from the private sector to complement government’s efforts. Additionally, strengthening community participation could improve vaccine acceptance. Lastly, the opportunities provided by social media should be harnessed to reach wider audience with accurate information on vaccines and immunisation to improve uptake. Media monitoring software could be installed to fish out rumours for prompt actions. Proactively engagement of the public on social media platforms could be enhanced through sharing of concise audiovisuals to generate discussions and strengthen uptake  of accurate information. This can potentially mitigate the spread of misinformation and disinformation about the malaria vaccine.

## Supplementary Information

Additional file 1. Adapted NVI checklist for evaluation of MVIP, Ghana; 2019-2021

## Data Availability

The data that support the findings of this study are available from the Ghana Health Service, but restrictions apply to the availability of these data, which were used under license for the current study, and so are not publicly available. Data are however available from the authors upon reasonable request and with permission of the Director General of the Ghana Health Service.

## Abbreviations

AEFI: Adverse event following immunization
AESI: Adverse event of special interest
CCEOP: Cold chain equipment optimization platform
DHIMS: District health information system
EPI: Expanded programme on immunization
FDA: Food and drug authority
GAVI: Global alliance for vaccines and immunization
GHS: Ghana health service
ICC: Interagency coordinating committee
IPTp: Intermittent preventive treatment in pregnancy
IRS: Indoor residual spraying
LLIN: Long-lasting insecticidal treated nets
LSM: Larval source management
MVIP: Malaria vaccine implementation programme
NITAG: National immunization technical advisory group
NVI: New vaccine introduction
PIE: Post-introduction evaluation
SMC: Seasonal malaria chemoprevention
VPD: Vaccine-preventable disease
