# Supplementary material for: An assessment of Ghana’s pilot of the RTS,S malaria vaccine implementation programme; 2019–2021: a retrospective study
Source: Malar J. 2024 Sep 27;23:290. doi: 10.1186/s12936-024-05113-8 (PMC11438052; doi:10.1186/s12936-024-05113-8)
Supplement: Supplementary file 1 — Additional file 1. Adapted NVI checklist for evaluation of MVIP, Ghana; 2019-2021 [file 12936_2024_5113_MOESM1_ESM.docx]

Table 2: Adapted WHO checklist for assessment of new vaccine introduction

| **SNo** | **Thematic area** | **Key indicators** | **Score** | | |
| --- | --- | --- | --- | --- | --- |
|  |  |  | **Planning**  **(0 or 1)** | **Implementation (0 or 1)** | **Composite (Total)** |
| 1 | Programme objective(s) | 1. Defined objective(s) of the vaccine introduction |  |  |  |
| 2 | Target population and delivery strategy | 1. Defined target population for the vaccine |  |  |  |
|  |  | 2. Availability of roll out strategy (country-wide versus phased) |  |  |  |
|  |  | 3. Strategy for reaching underserved populations in the target areas |  |  |  |
| 3 | Policy | 1. Revision of vaccination schedule to include malaria vaccine |  |  |  |
| 4 | Financial considerations | 1. Mobilization of local resources to support vaccine roll out |  |  |  |
| 5 | National coordination mechanisms | 1. Establishment of steering committee and technical sub-committees to oversee vaccine roll out |  |  |  |
| 6 | Cold chain, logistics, and vaccine management | 1. Updated cold chain capacity to accommodate the added vaccine |  |  |  |
| 7 | Waste management strategy | 1. Updated waste management capacity to accommodate anticipated increase in immunization waste |  |  |  |
| 8 | Monitoring and evaluation | 1. Monitoring and supervision of service delivery |  |  |  |
|  |  | 2. Conduct of PIE 6-12 months after vaccine introduction |  |  |  |
| 9 | Health worker training | 1. Training of existing health workers prior to roll out |  |  |  |
|  |  | 2. Availability of mechanisms for continuous capacity building |  |  |  |
| 10 | Pharmacovigilance | 1. Availability of safety monitoring mechanism |  |  |  |
| 11 | Advocacy, communication, and social mobilization | 1. Involvement of local leaders in advocacy |  |  |  |
|  |  | 2. Development of IEC materials |  |  |  |
|  |  | 3. Use of social media for mobilization and demand generation |  |  |  |
|  |  | 4. Launch of vaccine introduction |  |  |  |
|  |  | 5. Proactive communication mechanisms to address rumours and hesitancy |  |  |  |
| Total score |  | |  |  |  |

PIE=Post Introduction Evaluation IEC=Information, Education, and Communication
